# Phase II clinical trial to study the safety and efficacy of combined S-1 + oxaliplatin therapy as neoadjuvant chemotherapy for locally advanced gastric cancer in older patients

**DOI:** 10.1007/s10147-023-02373-3

**Published:** 2023-06-27

**Authors:** Mitsuhiko Ota, Hiroshi Saeki, Hideo Uehara, Yoshiko Matsuda, Satoshi Tsutsumi, Tetsuya Kusumoto, Hisateru Yasui, Yasunari Ubukata, Shohei Yamaguchi, Hiroyuki Orita, Naoki Izawa, Saburo Kakizoe, Mototsugu Shimokawa, Tomoharu Yoshizumi, Yoshihiro Kakeji, Masaki Mori, Eiji Oki

**Affiliations:** 1 Department of Surgery and Science, Graduate School of Medical Sciences, Kyushu University, Fukuoka, Japan; 2 Department of General Surgical Science, Graduate School of Medicine, Gunma University, 3-39-22, Showa-machi, Maebashi, Gunma 371-8511 Japan; 3 Department of Gastroenterological Surgery, National Hospital Organization Kyushu Cancer Center, Fukuoka, Japan; 4 Division of Gastrointestinal Surgery, Department of Surgery, Graduate School of Medicine, Kobe University, Kobe, Japan; 5 Department of Surgery, Oita Prefectural Hospital, Oita, Japan; 6 Department of Gastroenterological Surgery and Clinical Research Institute Cancer Research Division, National Kyushu Medical Center, Fukuoka, Japan; 7 Department of Medical Oncology, Kobe City Medical Center General Hospital, Kobe, Japan; 8 Department of General Surgical Science, Graduate School of Medicine, Gunma University, Maebashi, Japan; 9 Department of Surgery, Hiroshima Red Cross Hospital & Atomic Bomb Survivors Hospital, Hiroshima, Japan; 10 Department of Surgery, Nakatsu Municipal Hospital, Nakatsu, Japan; 11 Department of Clinical Oncology, St. Marianna University School of Medicine, Kawasaki, Japan; 12 Department of Surgery, Ilikai Medical INC Kakizoe Hospital, Hirado, Japan; 13 Department of Biostatistics, Yamaguchi University Graduate School of Medicine, Ube, Japan; 14 Tokai University School of Medicine, Isehara, Japan

**Keywords:** Gastrectomy, Gastric Cancer, Neoadjuvant Chemotherapy, Older Person, Clinical Trial, Phase 2

## Abstract

**Background:**

Gastrectomy with D2 dissection and adjuvant chemotherapy is the standard treatment for locally advanced gastric cancer (LAGC) in Asia. However, administering chemotherapy with sufficient intensity after gastrectomy is challenging. Several trials demonstrated the efficacy of neoadjuvant chemotherapy (NAC). However, limited studies explored the feasibility of NAC-SOX for older patients with LAGC. This phase II study (KSCC1801) evaluated the safety and efficacy of NAC-SOX in patients with LAGC aged ≥ 70 years.

**Methods:**

Patients received three cycles of SOX_130_ (oxaliplatin 130 mg/m^2^ on day 1, oral S-1 40–60 mg twice daily for two weeks every three weeks) as NAC, followed by gastrectomy with lymph node dissection. The primary endpoint was the dose intensity (DI). The secondary endpoints were safety, R0 resection rate, pathological response rate (pRR), overall survival, and relapse-free survival.

**Results:**

The median age of 26 enrolled patients was 74.5 years. The median DI in NAC-SOX_130_ was 97.2% for S-1 and 98.3% for oxaliplatin. Three cycles of NAC were administered in 25 patients (96.2%), of whom 24 (92.3%) underwent gastrectomy with lymphadenectomy. The R0 resection rate was 92.3% and the pRR (≥ grade 1b) was 62.5%. The major adverse events (≥ grade 3) were neutropenia (20.0%), thrombocytopenia (11.5%), anorexia (11.5%), nausea (7.7%), and hyponatremia (7.7%). Postoperative complications of abdominal infection, elevated blood amylase, and bacteremia occurred in one patient each. Severe diarrhea and dehydration caused one treatment-related death.

**Conclusions:**

NAC-SOX_130_ is a feasible therapy for older patients, although systemic management and careful monitoring of adverse events are necessary.

**Supplementary Information:**

The online version contains supplementary material available at 10.1007/s10147-023-02373-3.

## Introduction

According to the 2020 Global Cancer Observatory Data, gastric cancer (GC) is the 4th most deadly cancer in the world, and most common in East Asia [1]. Following lung and colorectal cancer, GC is the third leading cause of cancer-related death in Japan [2]. With a progressively aging population in Japan, the incidence of GC in older patients is increasing. Additionally, the same trend is being observed in China, Korea, and Taiwan. Most clinical trials aimed at developing standard therapies have involved patients with a median age of 65 years or less [3–5], and it is not clear whether the treatments can be similarly adapted to older patients, including vulnerable populations.

Gastrectomy with D2 lymph node dissection and postoperative adjuvant chemotherapy is currently the standard treatment for locally advanced GC (LAGC) in Asian countries [6–10]. However, the efficacy of this standard treatment is unsatisfactory, and administering chemotherapy with sufficient intensity after gastrectomy is often challenging [11–14]. In Europe and the United States, the phase III MAGIC and FLOT4-AIO studies showed the efficacy of neoadjuvant chemotherapy (NAC) for overall survival (OS) in patients with resectable GC/esophagogastric junction cancer (EGJC)/lower esophageal adenocarcinoma; thus, NAC has become the standard therapy [15, 16]. Additionally, the superiority of NAC has been reported in East Asia [17, 18]. S-1 plus oxaliplatin (SOX) therapy has a high response rate for advanced GC [19]. We have been developing preoperative SOX therapy (KSCC1601) and have reported SOX_130_ (oxaliplatin 130 mg/m^2^ on day 1, oral S-1 40–60 mg twice daily for two weeks every three weeks) and demonstrated its substantial benefit for LAGC and EGJC [20].

The current phase II study, named KSCC1801, was conducted to investigate the safety and efficacy of SOX_130_ as preoperative chemotherapy for LAGC patients aged 70 years or older. Through KSCC1801, we aimed to accumulate data on safety in older patients and expand the scope of NAC for Stage II/III GC. Once the safety and efficacy of NAC-SOX plus surgery are confirmed in this study, we will consider the patients and study arms for future studies.

## Patients and methods

This multicenter, open-label, single-arm, prospective phase II clinical trial was conducted from June 2018 to May 2020 at 11 institutions in Japan. The study protocol was approved by the Clinical Research Network Fukuoka Certified Review Board. The study was conducted according to the tenets of the Declaration of Helsinki and the Clinical Trials Act. Written informed consent was obtained from all eligible patients prior to registration. The study protocol was registered in the Japan Registry of Clinical Trials (https://jrct.niph.go.jp) as jRCTs071180001.

### Eligibility criteria

All patients had histologically confirmed untreated gastric adenocarcinoma based on an endoscopic biopsy of the primary lesion. The major inclusion criteria were diagnoses of cT3–4, N1–3, and M0 (according to the Japanese Gastric Cancer Classification: 3rd English edition) based on image findings (endoscopy, abdominal CT), and laparoscopically proven H0, P0, CY0. In addition, patients who were capable of oral intake were Eastern Cooperative Oncology Group performance status (ECOG-PS) ≤ 1 and over 70 years of age. Exclusion criteria included an esophageal infiltration distance of 3 cm or more, presence of liver cirrhosis or active hepatitis, history of neurologic or psychiatric disorders, cardiovascular disease, drug hypersensitivity, or another cancer diagnosis within the past five years. Detailed inclusion and exclusion criteria are listed in Online Resource 1.

### Treatment protocol

Patients received three courses of SOX_130_ continuously as NAC, followed by gastrectomy for curative purposes. Protocol treatment was considered complete when both preoperative chemotherapy and surgery completion, as described below, were met. However, if the preoperative chemotherapy was discontinued before completing three courses of the regimen, and the patient met the “preoperative re-evaluation criteria” and underwent gastrectomy, protocol treatment was considered complete. Treatment after completion and discontinuation of protocol treatment (e.g., postoperative adjuvant therapy) was not specified.

### Neoadjuvant SOX chemotherapy

Patients were scheduled to receive three courses of NAC with oxaliplatin (130 mg/m^2^) by intravenous infusion on Day 1 and oral S-1 (twice daily) for 14 days, repeated every three weeks. The dose of S-1 was determined by body surface area (BSA) and creatinine clearance (CCr). For CCr 50 mL/min and above, the dose was 80 mg/day for BSA < 1.25 m^2^, 100 mg/day for 1.25 m^2^ ≤ BSA < 1.5 m^2^, and 120 mg/day for BSA ≥ 1.5 m^2^. In CCr 50–40 mL/min, S-1 was used with a one-step reduction. The NAC discontinuation criteria are provided in Online Resource 2. The preoperative evaluation was performed after the final course of treatment. NAC was considered complete with the administration of the oxaliplatin dose in the third course.

### Surgery

Since the current clinical trial was for older patients, the protocol surgery was only stipulated as “surgery for curative purposes.” Surgery was performed after confirming that the following two conditions were met. (1) A CT scan performed within 7–56 days from the date of the last S-1 administration to determine that surgery for curative purposes is feasible. (2) The patient must have a white blood cell count ≥ 3,000/mm^3^ and platelet count ≥ 75,000/mm^3^ on the most recent laboratory test performed within 14 days prior to surgery. Gastrectomy with lymphadenectomy was performed following standard procedures. The surgery was considered complete if the above criteria were met and if the surgery was performed within 56 days from the last day of S-1 administration. Even if NAC was discontinued before the completion of three courses, surgery was executed if preoperative re-evaluation criteria were met.

### Post-operative evaluation

From the end of surgery to 30 days after surgery, the items that were evaluated included: (1) Initial discharge date after surgery; (2) Presence or absence of postoperative hemorrhage; (3) Presence or absence of re-operation; (4) Pathological findings; and (5) Postoperative early complications. The principal investigator’s or sub-investigator’ judgment related to the causal relationship with surgery was also recorded. Assessment of surgical complications was performed according to the Common Terminology Criteria for Adverse Events (CTCAE v4.0) and Clavien–Dindo classification.

### Statistical analysis

The primary endpoint of the trial was the dose intensity (DI) of the preoperative SOX_130_ therapy. Following a previous report [21], the DI was defined as the cumulative dose of each drug that was administered (mg)/cumulative dose that would be administered when three courses of treatment are completed without drug holiday or dose reduction (planned dose) (mg) × 100 (%). Since the participants in this study were older patients and the incidence of chemotherapy-related adverse events was estimated to be higher than in younger patients, the primary endpoint was an assessment of the tolerability of chemotherapy. Based on previous studies, this study adopted a DI threshold of 75% for both S-1 and oxaliplatin in NAC-SOX_130_, with an expected value of at least 85% considered clinically significant and a standard deviation (SD) of 11–14% [21–24]. In the main analysis, a one-sided significance level of 2.5% was used for each of S-1 and oxaliplatin, and if both were statistically significant, the study treatment was effective. To achieve a power of 80% for the primary analysis, a power of 90% for each test would require 23 cases. Assuming some losses based on patient ineligibility or dropout, the target number of cases was 25. The tolerability index in this study was preoperative SOX_130_ therapy DI. The rate of protocol treatment completion and relative dose intensity (RDI) were set as secondary endpoints as supportive data for the evaluation of tolerability. Additionally, overall survival (OS), relapse-free survival (RFS), pathological response rate (pRR), and R0 resection rate were defined as secondary endpoints to evaluate efficacy. Statistical analysis was performed using SAS version 9.4 (SAS Institute, Cary, NC, USA).

## Results

### Patient characteristics

Twenty-six patients were enrolled in this study. Their median age was 74.5 years (range: 70–82); 73.1% were men. Their ECOG-PS scores were 0: 57.7%, and 1: 42.3%. Eighteen cases (69.2%) were of the differentiated type. Sixteen patients (61.5%) had T4 tumors, and 11 patients (42.3%) had N2 lymph nodes. Table 1 summarizes their baseline characteristics. Considering the patient population, the Geriatric 8 (G8) score was measured to assess patient frailty. The median G8 score was 12.25. To standardize the evaluation of older patients, the Charlson Comorbidity Index was used. The mean value of the Charlson Comorbidity Index was 0.3 (SD ± 0.61).

**Table 1:** Patient characteristics

Variable	Patients, (N = 26)
	n (%)
Sex	
Men	19(73.1)
Women	7(26.9)
	Median (range)
Age	74.5(70–82)
	n (%)
ECOG-PS	
0	15(57.7)
1	11(42.3)
	Median (range)
G8	12.25(8–16)
	Mean ± SD
Charlson comorbidity index	0.3 ± 0.61
	n (%)
Histological type of gastric cancer	
Differentiated	18(69.2)
Undifferentiated	8(30.8)
cT Stage^a^	
T3	10(38.5)
T4a	14(53.8)
T4b	2(7.7)
cN stage^a^	
N1	15(57.7)
N2	11(42.3)
Gastric cancer HER2 protein (IHC)	
0	2(7.7)
1 +	5(19.2)
2 +	1(3.8)
3 +	1(3.8)
Unknown	17(65.4)

*ECOG-PS* Eastern Cooperative Oncology Group Performance Status, *G8* Geriatric 8, *cT* clinical T grade, *cN* clinical N grade, *IHC* Immunohistochemistry

^a^According to Japanese Gastric Cancer Classification: 3rd English edition

### Neoadjuvant chemotherapy

**Fig. 1:**
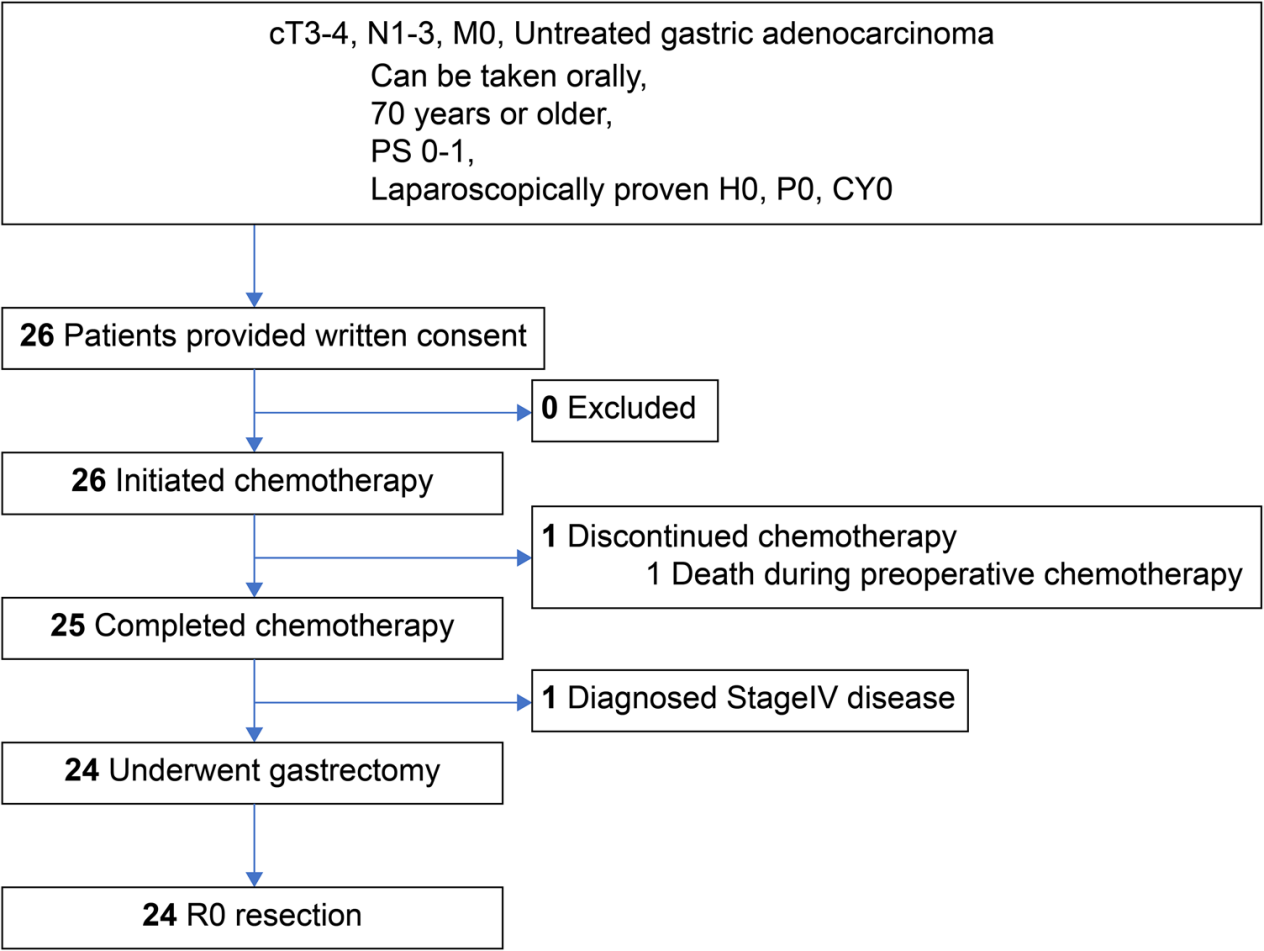
CONSORT flow diagram

Twenty-five patients (96.2%) completed three cycles of preoperative SOX_130_ therapy (Fig. 1). The median NAC-SOX_130_ DI was 97.2% for S-1 (95% CI: 83.1–96.2) and 98.3% (95% CI: 85.7–97.3) for oxaliplatin (Table 2). In this study, we adopted 75% as the threshold DI for both S-1 and oxaliplatin in NAC-SOX_130_ for patients over 70 years old; the expected value was set to 85% or more (clinically significant) and the SD was set to 11 to 14%. As the values obtained in our study were higher than the predefined age-adjusted threshold values for S-1 and oxaliplatin, our study therefore met its primary endpoint. The mean RDI in NAC-SOX_130_ for S-1 was 82.1% ± 13.08 and was 84.4% ± 13.44 for oxaliplatin (Table 2). The clinical response rates to NAC were evaluated in the full analysis set. Clinical complete response was achieved in one patient (10%), and six patients (60%) showed partial response; hence, the response rate was 70% (Online Resource 3).

**Table 2:** Dose intensity and Relative dose intensity of neoadjuvant three courses SOX_130_ therapy

	N	Mean (%)	SD	Median (%)	Min (%)	Max (%)	95% CI	P value
DI								
Oxaliplatin	26	91.5	14.28	98.3	33.3	104.4	85.7–97.3	P < 0.0001
S-1	26	89.6	16.12	97.2	29.8	101.2	83.1–96.2	P < 0.0001
RDI								
Oxaliplatin	26	84.4	13.44	83.8	54.4	102.8		
S-1	26	82.1	13.08	82.7	57.5	101.2		

*DI* Dose intensity, *RDI* Relative dose intensity, *CI* Confidence intervals

The incidence of adverse events is presented in Table 3. The major adverse events (≥ grade 3) were neutropenia (20.0%), thrombocytopenia (11.5%), and hyponatremia (7.7%) in hematological toxicity, and anorexia (11.5%), nausea (7.7%), and fatigue (7.7%) in non-hematological toxicity. All patients had at least one adverse event. One treatment-related death occurred, probably owing to severe diarrhea and dehydration. The patient was inducted into the first course of chemotherapy in an inpatient setting. The patient had diarrhea (Grade 2) since Day 11 and was on an outpatient IV infusion. However, on Day 13, the patient was transported to the emergency room due to dehydration and subsequently died.

**Table 3:** Adverse events during the neoadjuvant SOX_130_ therapy

Toxicities (N = 26)	Grade 3–4, n (%)	All grades (1–4), n (%)
Objective findings		
Anorexia	3 (11.5)	22 (84.6)
Constipation	0 (0.0)	8 (30.8)
Dehydration	1 (3.8)	1 (3.8)
Diarrhea	0 (0.0)	9 (34.6)
Enterocolitis	1 (3.8)	1 (3.8)
Eye disorders	0 (0.0)	1 (3.8)
Fatigue	2 (7.7)	8 (30.8)
Febrile neutropenia	1 (3.8)	1 (3.8)
Fever	1 (3.8)	5 (19.2)
Gastric hemorrhage	1 (3.8)	1 (3.8)
Infections and infestations	1 (3.8)	2 (7.7)
Malaise	0 (0.0)	15 (57.7)
Mucositis oral	0 (0.0)	3 (11.5)
Nausea	2 (7.7)	16 (61.5)
Peripheral sensory neuropathy	0 (0.0)	19 (73.1)
Respiratory, thoracic, and mediastinal disorders	1 (3.8)	1 (3.8)
Skin and subcutaneous tissue disorders	0 (0.0)	1 (3.8)
Skin hyperpigmentation	0 (0.0)	3 (11.5)
Vomiting	0 (0.0)	4 (15.4)
Watering eyes	0 (0.0)	2 (7.7)
Palmar-plantar erythrodysesthesia syndrome	0 (0.0)	4 (15.4)
Laboratory findings		
Leucopenia	2 (7.7)	10 (38.5)
Neutropenia	5 (20.0)	19 (76.0)
Thrombocytopenia	3 (11.5)	21 (80.8)
Anemia	1 (3.8)	26 (100)
Aspartate aminotransferase increased	0 (0.0)	18 (69.2)
Alanine aminotransferase increased	0 (0.0)	12 (46.2)
Hypoalbuminemia	0 (0.0)	26 (100)
Hypernatremia	0 (0.0)	3 (11.5)
Hyponatremia	2 (7.7)	11 (42.3)
Hyperkalemia	1 (3.8)	6 (23.1)
Hypokalemia	0 (0.0)	5 (19.2)
Creatinine increased	0 (0.0)	4 (15.4)

### Surgical and pathological findings

Of the 25 patients who completed preoperative chemotherapy, 24 patients received gastrectomy, and all underwent R0 resection (R0 resection rate = 92.3%; Fig. 1, Table 4). One patient did not undergo gastrectomy due to disease progression. Gastrectomy was performed laparoscopically in more than half of the patients, 54% were total gastrectomy, and all cases had at least D2 lymph node dissection. There were no intraoperative Grade 3 or higher complications. The pRR in eligible patients was 62.5% (95% CI 40.6–81.2) including marked response in four patients (16.7%, Table 5). These postoperative complications were observed in one case each (3.8%): grade 4 elevated blood amylase, grade 3 bacteremia, and grade 2 abdominal infection, Postoperative adjuvant therapy (not specified in the protocol) was administered to 18 patients (75%); the regimens were S-1 monotherapy in 15 patients, SOX in one patient, and docetaxel plus S-1 in two patients.

**Table 4:** Surgical and postoperative findings

Variables	Patients, n (%)
Surgical approach	
Open	11 (45.8)
Laparoscopy	13 (54.2)
Type of gastrectomy	
Total gastrectomy	13 (54.2)
Distal gastrectomy	10 (41.7)
Proximal gastrectomy	1 (4.2)
Combined resection	
No	17 (70.8)
Done	7 (29.2)
Spleen/gall bladder/liver (duplicated)	2/5/1 (8.3/20.8/4.2)
LN dissection	
D2	23 (95.8)
D2 +	1 (4.2)
Reconstruction	
Billroth-I	4 (16.7)
Roux-en Y	19 (79.2)
Double tract	1 (4.2)
Residual tumor	
R0	24 (100)
Intraoperative complication (Grade3 ≤)	0 (0)
Postoperative complication (Grade3 ≤)	2 (8.3)
Adjuvant chemotherapy	
No	6 (25)
Done	18 (75)

**Table 5:** Pathological findings

	N = 24	95% CI
	n (%)	
pT Stage^a^		
T0	3 (12.5)	
T1a	0 (0)	
T1b	1 (4.2)	
T2	5 (20.8)	
T3	14 (58.3)	
T4a	1 (4.2)	
pN Stage^a^		
N0	10 (41.7)	
N1	5 (20.8)	
N2	5 (20.8)	
N3a	3 (12.5)	
N3b	1 (4.2)	
pStage^a^		
Stage IA	2 (8.3)	
Stage IB	1 (4.2)	
Stage IIA	8 (33.3)	
Stage IIB	3 (12.5)	
Stage IIIA	6 (25.0)	
Stage IIIB	3 (12.5)	
Stage IIIC	1 (4.2)	
Pathological response		
Grade 0	1 (4.2)	
Grade 1a	8 (33.3)	
Grade 1b	7 (29.2)	
Grade 2	4 (16.7)	
Grade 3	4 (16.7)	
pRR^b^	15 (62.5)	40.6–81.2

^a^According to Japanese Gastric Cancer Classification: 3rd English edition

^b^pRR: pathological response rate; was defined as the ratio of grade 1b–3 primary tumors; *CI* Confidence intervals

### Survival

**Fig. 2:**
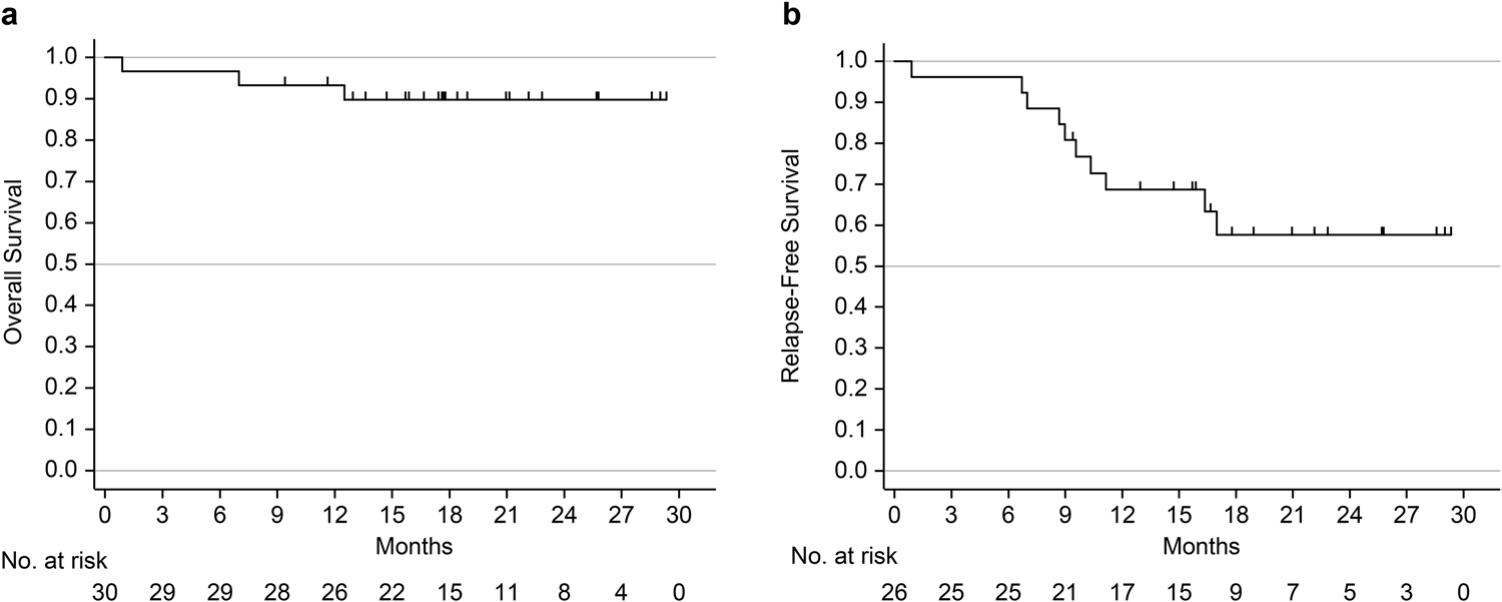
**a** Overall 1- and 2-year survival rates. **b** The relapse free survival rates (RFS) for 1 year and 2 years

There were three deaths during the observation period, including one progressive disease, one treatment-related death, and one death from other causes. The 1- and 2-year OS rates were 93.3% and 89.7%, respectively (Fig. 2A). Eight recurrences were observed during the observation period. The 1- and 2-year RFS rates were 68.7% and 58.6%, respectively (Fig. 2B).

## Discussion

This phase II study tested the safety and efficacy of SOX_130_ therapy as preoperative chemotherapy followed by radical gastrectomy in older LAGC patients. The primary endpoint–DI–was 97.2% for S-1 and 98.3% for oxaliplatin. Although adverse events should be monitored, NAC-SOX_130_ followed by surgery was shown to be an acceptable treatment strategy for older LAGC patients.

The main research hypothesis of this study was that “NAC + radical gastrectomy for LAGC in older patients is as safe and effective as in the young.” The participants in this study were older than 70 years of age, were more likely to have comorbidities, and the incidence of adverse events during chemotherapy was expected to be higher than in younger patients. Therefore, we focused on safety evaluation, and set the DI of the preoperative SOX_130_ therapy as the primary endpoint. The median DI in this study was oxaliplatin 91.5% (95% CI 85.7–97.3) and S-1 89.6% (95% CI 83.1–96.2). With regards to the SOX_100_ therapy, which uses an oxaliplatin dose of 100 mg/m^2^, Shitara et al. reported on its use as adjuvant chemotherapy in patients who underwent gastrectomy with D2 dissection, and mentioned that among the 31 patients aged 65 years or older, the RDI was 77.1% for S-1 and 71.7% for oxaliplatin [22]. Furthermore, Bando et al. carried out an analysis by age group in the G-SOX study involving patients with unresectable/recurrent GC and reported a median RDI of 74.12% for S-1 and 75.00% for oxaliplatin among patients who were 70 years or older [23]. Compared to these previous reports, the RDI for SOX_130_ therapy was maintained in the KSCC1801 study. This is consistent with the fact that preoperative chemotherapy is generally more likely to maintain the RDI compared to the postoperative or metastatic setting. On the other hand, in the NAC setting, the RDI for three courses of SOX_130_ is reported to be as high as 91.6% for S-1 and 92.1% for L-OHP [24]. However, this study included only 14 patients with an upper age limit of 70 years, and the results cannot be simply compared with those of the current study of older patients. A subgroup analysis of the CRITICS trial, which examined the benefit of perioperative chemotherapy, reported a lower RDI for preoperative/postoperative chemotherapy in older patients compared to younger patients [25]. Compared to these previously reported SOX therapies, NAC-SOX_130_ could be administered at a sufficient therapeutic intensity in older patients.

In three trials of preoperative chemotherapy for stage III disease, the R0 resection rate was over 90% and the pRR was over 40% [24, 26, 27]. Hosoda et al. conducted the KDOG1001 study evaluating the effect of adding docetaxel to preoperative CS (cisplatin + S-1) therapy (DCS therapy), with an R0 resection rate of 90% and pRR of 57.5% in the DCS group. In terms of NAC adverse events, neutropenia of grade 3 or greater was more common in the DCS group (55%) than in the CS group (29%) [28]. A phase II trial (OGSG1601) of perioperative CapeOX (capecitabine + oxaliplatin) was conducted in patients with clinical SS/SE N1–3 GC. In perioperative CapeOX therapy, the R0 resection rate was 78.4% with a pRR of 54.1%. Preoperative chemotherapy grade 3 or higher adverse events were neutropenia, thrombocytopenia, and anorexia each observed in 8% of the cohort [29]. In the current study, the R0 resection rate was 92.3% and the pRR was 62.5%, confirming numerical equivalent efficacy to DCS. In the KSCC1601 study of NAC-SOX_130_ for advanced GC including EGJC in patients 20 years and older, the R0 resection rate was 87.2% and the pRR was 59.5% [20]. We consider NAC-SOX_130_ therapy effective in older patients. Postoperative complications and prognoses were comparable to previous reports.

All Grade or Grade 3–4 adverse events in preoperative chemotherapy in the older patients in this study were neutropenia and hyponatremia in 76.0/20.0% and 42.3/7.7%, respectively. We experienced one case of death due to severe diarrhea and dehydration. In KSCC1601 discussed above, which included patients aged 20 years and older, these adverse events were lower at 42.6/6.4% and 29.8/2.1%, respectively, suggesting that these adverse events may be more frequent and severe in older patients. We lost one patient due to diarrhea and dehydration. This may have been avoided if the patient had been hospitalized immediately after the onset of diarrhea. In the case of elderly patients, it is considered safe to hospitalize them when the first course of NAC-SOX therapy is introduced. Since the older patients have decreased organ function, careful observation should be continued even after outpatient follow-up, and hospitalization should be considered depending on the patient’s condition.

There are various reports on the significance of chemotherapy in older patients with advanced or recurrent GC, although the issue remains controversial. This is because the older population is diverse, and patients cannot be stratified by age alone. A phase II study of S-1 monotherapy in patients aged 75 years or older reported favorable safety and efficacy [30]. Another report found no difference in survival between S-1 and CS therapy in a retrospective analysis of 58 patients aged 70 years or older with unresectable or recurrent GC [31]. A randomized phase III trial in the United Kingdom examining the treatment efficacy and quality of life of CapeOX therapy in older or frail gastroesophageal cancer patients reported that reduced-intensity chemotherapy offered a superior patient experience without significantly compromising cancer control [32]. In addition, geriatric assessment helps in predicting the utility of chemotherapy. On the other hand, a randomized phase III trial was conducted in Korea comparing capecitabine monotherapy with CapeOX as standard therapy for the first-line treatment of metastatic GC patients over 70 years old. In the first interim analysis, the median OS was better with CapeOX (11.1 months) than with single-agent capecitabine (6.3 months), although with no significant difference, and the independent data monitoring committee recommended discontinuation of the trial [33]. This trial was conducted in older patients with PS 0–2 and preserved organ function, suggesting that standard treatment is feasible and effective in this population. A review of older patients with advanced recurrent esophagogastric adenocarcinoma also reported that older, fit patients can be treated in the same way as younger patients [34]. Thus, the older population includes patients who can benefit from standard chemotherapy and those for whom diminishing treatment intensity improves the patient’s quality of life while maintaining therapeutic efficacy. Patients enrolled in the KSCC1801 study ranged in age from 70 to 82 years, with a median G8 of 12.25; NAC-SOX_130_ therapy was effective in this population, but it was not a population that could be treated at the same risk as younger patients due to the potential for increased adverse events. In a gastric cancer clinical trial including older patients, SOX therapy was administered to patients up to 89 years of age, and age alone cannot determine the therapeutic limit of SOX therapy [20, 23, 35, 36]. In terms of adverse events, renal function has been reported to be associated with chemotherapy-related adverse events in older cancer patients [37]. It is also known that S-1 administration in patients with impaired renal function may decrease renal excretion of the fluorouracil catabolite inhibitor, gimerasil, resulting in increased blood fluorouracil concentrations and more severe adverse effects. In older patients undergoing SOX therapy, renal function should be given particular attention among the age-related declines in organ function. As the aging population increases, there is an urgent need to develop stratification tools to provide precision medicine and help inform clinical decision making for older patients with LAGC undergoing preoperative chemotherapy [17, 18].

Another issue is the need for postoperative adjuvant chemotherapy after NAC. In the KSCC1801 study of older patients, postoperative adjuvant chemotherapy was not specified in the protocol due to safety concerns. However, 75% of the patients received postoperative adjuvant chemotherapy at the physician’s discretion. In Europe and the United States, perioperative chemotherapy is the standard treatment for locally advanced gastric cancer (NAC + surgery + postoperative chemotherapy). In East Asia, surgery plus postoperative adjuvant chemotherapy has been the standard treatment, and NAC’s additional effect has been verified. It remains unclear whether postoperative adjuvant chemotherapy can be omitted when NAC is administered, and whether there is a group of patients for whom it can be omitted. Subgroup analyses of clinical trials of postoperative adjuvant chemotherapy have not demonstrated an additional OS benefit in older patients [7, 9], and the significance of postoperative adjuvant chemotherapy in elderly patients is unclear. The present study demonstrated that NAC is feasible in older patients. Therefore, whether NAC–which is generally expected to be better tolerated than postoperative adjuvant chemotherapy–can be an alternative to postoperative adjuvant chemotherapy in older patients with gastric cancer requires further investigation.

There were a few limitations to the study. First, the study was a single-arm, phase II clinical trial involving a small number of patients. Second, no comprehensive geriatric assessment or quality of life assessment was included in the study design.

## Conclusion

In this trial, the safety and efficacy of NAC-SOX_130_ followed by surgery for LAGC in older patients were confirmed. Systemic management and more careful monitoring of adverse events are necessary for older patients. The results suggest that preoperative chemotherapy with NAC-SOX_130_ may be a treatment option for older patients with LAGC, although further validation in phase III trials is required.

## Supplementary Information

Below is the link to the electronic supplementary material.

Supplementary file1 (PDF 422 KB)

Supplementary file2 (PDF 289 KB)

Supplementary file3 (PDF 466 KB)
