# Supplementary material for: Phase II clinical trial to study the safety and efficacy of combined S-1 + oxaliplatin therapy as neoadjuvant chemotherapy for locally advanced gastric cancer in older patients
Source: Int J Clin Oncol. 2023 Jun 27;28(9):1166–75. doi: 10.1007/s10147-023-02373-3 (PMC10468941; doi:10.1007/s10147-023-02373-3)
Supplement: Supplementary file 1 — Supplementary file1 (PDF 422 KB) [file 10147_2023_2373_MOESM1_ESM.pdf]

Phase II Clinical Trial to Study the Safety and Efficacy of Combined S-1 + Oxaliplatin Therapy as Neoadjuvant Chemotherapy for Locally Advanced Gastric Cancer in Older Patients

Mitsuhiko Ota, Hiroshi Saeki, Hideo Uehara, Yoshiko Matsuda, Satoshi Tsutsumi, Tetsuya Kusumot, Hisateru Yasui, Yasunari Ubukata, Shohei Yamaguchi, Hiroyuki Orita, Naoki Izawa, Saburo Kakizoe, Mototsugu Shimokawa, Tomoharu Yoshizumi, Yoshihiro Kakeji, Masaki Mori & Eiji Oki

Corresponding author:

Hiroshi Saeki, MD, PhD,
Department of General Surgical Science, Gunma University Graduate School of Medicine
E-mail: h-saeki@gunma-u.ac.jp

Online Resource: Supplementary Table 1. Inclusion and exclusion criteria

Inclusion criteria	1) Capable of providing written consent to participate in the research and receive treatment based on this protocol.<br>2) Histologically confirmed gastric adenocarcinoma (one of the general type histological classifications, or lymphocyte-infiltrating gastric cancer) based on endoscopic biopsy of the primary lesion.<br>3) Diagnosed as cT3–4 based on image findings (endoscopy, abdominal contrast-enhanced CT).<br>4) Upper abdominal contrast-enhanced CT shows swollen lymph nodes in the gastric region with a minor axis of 8 mm or more, or a major axis of 10 mm or more. However, the patient may be enrolled even when these requirements are not met, if many lymph nodes are swollen and the clinical diagnosis is clearly positive for lymph node metastasis （N1–3）.<br>5) None of the following metastases are evident based on the thoracic contrast-enhanced, upper abdominal contrast-enhanced, and pelvic contrast-enhanced taken within 28 days prior to enrollment.<br>a. Pulmonary metastasis<br>b. Peritoneal metastasis<br>c. Hepatic metastasis<br>d. Pleural effusion, ascites beyond the pelvic cavity<br>e. Lymph node metastases other than in regional lymph nodes*<br>f. Other distant metastases considered M1 metastases<br>*Regional lymph nodes: Lymph node numbers 1–12 and 14v are regional lymph nodes of the stomach.<br><br>6) No clinical cervical lymph node metastasis/distant metastasis.<br>7) H0/P0 by laparoscopy (open-abdomen examination is allowed) conducted within 28 days prior to enrollment, and CY0 by peritoneal lavage cytology.<br>8) No residual gastric cancer.<br>9) Capable of oral ingestion.<br>10) No history of chemotherapy, radiotherapy and endocrine therapy within the previous 5 years, including treatments for other types of cancer.<br>11) Aged 70 years or older at the time of enrollment.<br>12) Eastern Cooperative Oncology Group performance status (ECOG-PS) of 0 or 1 (PS must be described in the patient chart)<br>13) The latest laboratory investigation parameters measured within 14 days prior to enrollment (can be up to the same day of the week, 2 weeks before the date of enrollment) satisfy the following:<br>a. Neutrophil count ≧ 1,500 /mm^3

	b. Hemoglobin ≧ 8.0 g/dL (no blood transfusions given within 14 days prior to the date of blood draws for tests used for enrollment）<br>c. Platelet count ≧ 10×104 /mm3<br>d. Total bilirubin ≦1.5 mg/dL<br>e. AST ≦ 100 U/L<br>f. ALT ≦ 100 U/L<br>g. Creatinine clearance** ≧ 40 mL/min<br>** The following formula was used to calculate the creatinine clearance (Cockcroft＆Gault’s formula). Measured creatinine clearance values are not used.<br>Male: (140 - Age) x Body weight (kg)/72 x Serum creatinine (mg/dL)<br>Female: 0.85 x Value calculated using the formula for male patients
Exclusion criteria	Exclusion criteria<br>1) Diagnosed with a distance of esophageal infiltration of 3 cm or more.<br>2) Type 4 gastric cancer that extends to the entire stomach macroscopically.<br>3) Have a serious drug hypersensitivity (particularly to platinum preparations, 5-FU, S-1).<br>4) Have peripheral sensory neuropathy (Grade 1 or higher) of the sensory system.<br>5) Have an infection requiring systemic treatments.<br>6) Have poorly-controlled hypertension.<br>7) Have diabetes that is poorly controlled by medication.<br>8) Have clinically problematic heart disease.<br>Congestive heart failure, angina requiring pharmacotherapy, transmural myocardial infarction seen clearly on ECG, clinically apparent cardiovascular disease, symptomatic coronary artery disease, poorly-controlled arrhythmia, and history of myocardial infarction developing during the previous 12 months<br>Having unstable angina (angina that developed or exacerbated during the previous 3 weeks), or a history of myocardial infarction during the previous 6 months.<br>9) Have a severe lung disease (such as interstitial pneumonia, pulmonary fibrosis, severe emphysema).<br>10) Clinically problematic psychiatric disorder or a history of central nervous system neuropathy.<br>11) Fresh bleeding from the gastrointestinal tract requiring repeated blood transfusion.<br>12) Receiving treatments with phenytoin, warfarin potassium, or flucytosine.<br>13) Have diarrhea symptoms (Grade 2 or higher).<br>14) Have an active double cancer.<br>Carcinoma in situ or lesions equivalent to intramucosal cancer, which are synchronous double cancer and metachronous double cancer with a disease-free period of 5 years or less and are deemed to be cured by local treatment are not regarded as active double cancers.<br>However, even if the disease-free period is less than 5 years, the history of clinical stage I prostate cancer and completely resected cancer of the following pathological stages are not regarded as active double/multiple cancers.<br>Gastric cancer “adenocarcinoma (general type)”: Stage 0–I; colon cancer (adenocarcinoma): Stage 0–I; esophageal cancer (squamous cell carcinoma, adenosquamous carcinoma, basal cell carcinoma): Stage 0; breast cancer (non-invasive ductal carcinoma in situ, non-invasive lobular cancer): Stage 0; breast cancer (invasive ductal carcinoma, invasive lobular carcinoma, Paget's disease): Stage 0–IIA; endometrial cancer (endometrial adenocarcinoma, mucinous adenocarcinoma): Stage I; prostate cancer (adenocarcinoma): Stage I–II, cervical cancer (squamous cell carcinoma): Stage 0; thyroid cancer (papillary carcinoma, follicular carcinoma): Stage I, II and III; gastric cancer (clear cell carcinoma, chromophobe cell carcinoma): Stage I.<br>15) Male patients who wish their partner to become pregnant.<br>16) Have liver cirrhosis or active hepatitis based on “Hepatitis B Treatment Guidelines” and “Hepatitis C Treatment Guidelines”<br>(https://www.jsh.or.jp/medical/guidelines/jsh_guidlines/hepatitis_b)<br>(https://www.jsh.or.jp/medical/guidelines/jsh_guidlines/hepatitis_c)<br>17) Deemed ineligible to participate in the study by the Principal Investigator or Sub investigators for other reason(s).
