# Supplementary material for: Phase II clinical trial to study the safety and efficacy of combined S-1 + oxaliplatin therapy as neoadjuvant chemotherapy for locally advanced gastric cancer in older patients
Source: Int J Clin Oncol. 2023 Jun 27;28(9):1166–75. doi: 10.1007/s10147-023-02373-3 (PMC10468941; doi:10.1007/s10147-023-02373-3)
Supplement: Supplementary file 2 — Supplementary file2 (PDF 289 KB) [file 10147_2023_2373_MOESM2_ESM.pdf]

**Phase II Clinical Trial to Study the Safety and Efficacy of Combined S-1 + Oxaliplatin Therapy as Neoadjuvant Chemotherapy for Locally Advanced Gastric Cancer in Older Patients**

Mitsuhiko Ota, Hiroshi Saeki, Hideo Uehara, Yoshiko Matsuda, Satoshi Tsutsumi, Tetsuya Kusumot, Hisateru Yasui, Yasunari Ubukata, Shohei Yamaguchi, Hiroyuki Orita, Naoki Izawa, Saburo Kakizoe, Mototsugu Shimokawa, Tomoharu Yoshizumi, Yoshihiro Kakeji, Masaki Mori & Eiji Oki

**Corresponding author:**

Hiroshi Saeki, MD, PhD,
Department of General Surgical Science, Gunma University Graduate School of Medicine,
e-mail: h-saeki@gunma-u.ac.jp

Online Resource: Electronic Supplementary Table 2. Neoadjuvant chemotherapy discontinuation criteria.

| | |
|---|---|
| If a patient meets any of the following criteria, the neoadjuvant chemotherapy for the said patient will be discontinued. If the use of one of the drugs will be terminated due to adverse events or “5.3.4. Dose reduction criteria,” consider discontinuing neoadjuvant chemotherapy and switching to surgery rather than continuing with a single-agent treatment with the other drug. | |
| Even if a patient stops receiving neoadjuvant chemotherapy, if he/she meets “5.3.7. Preoperative re-evaluation criteria,” he/she should switch to surgery and the protocol treatment is not discontinued. | |
| 1. | If neoadjuvant chemotherapy is deemed ineffective due to clear exacerbation of the primary disease during neoadjuvant chemotherapy an overall assessment including clinical findings should be made on whether or not the patient should transition to surgery, rather than based on image assessment alone. |
| 2. | *If neoadjuvant chemotherapy cannot be continued due to adverse events.<br>1) If Grade 3 or higher peripheral sensory neuropathy is confirmed<br>2) If Grade 3 or higher allergic reaction/hypersensitivity is confirmed<br>3) If the next course of treatment cannot be started within 28 days of the last dose of S-1 because the “Table 5.3.1.1 Criteria for the start of course” are not met due to an adverse event<br>4) If Grade 4 non-hematological toxicities are observed, except in the case of the following adverse events:<br>* ALP, γ-GTP, hyperglycemia, hypercalcemia, hypocalcemia, hypernatremia, hyponatremia, hyperkalemia, hypokalemia<br>5) If the Principal Investigator or Sub investigator(s) deem the need to discontinue neoadjuvant chemotherapy due to other adverse events |
| 3 | If a patient requests to discontinue neoadjuvant chemotherapy for reasons unrelated to adverse events. |

| | |
|---|---|
| 4 | If the patient dies during neoadjuvant chemotherapy |
| 5 | If the treatment is changed following the discovery that a patient is ineligible to participate in the study for other reasons, such as disease exacerbation after enrollment and before the start of treatment, or discovery of protocol violations. |
