# Supplementary material for: Phase II clinical trial to study the safety and efficacy of combined S-1 + oxaliplatin therapy as neoadjuvant chemotherapy for locally advanced gastric cancer in older patients
Source: Int J Clin Oncol. 2023 Jun 27;28(9):1166–75. doi: 10.1007/s10147-023-02373-3 (PMC10468941; doi:10.1007/s10147-023-02373-3)
Supplement: Supplementary file 3 — Supplementary file3 (PDF 466 KB) [file 10147_2023_2373_MOESM3_ESM.pdf]

Phase II Clinical Trial to Study the Safety and Efficacy of Combined S-1 + Oxaliplatin Therapy as Neoadjuvant Chemotherapy for Locally Advanced Gastric Cancer in Older Patients

Mitsuhiko Ota, Hiroshi Saeki, Hideo Uehara, Yoshiko Matsuda, Satoshi Tsutsumi, Tetsuya Kusumot, Hisateru Yasui, Yasunari Ubukata, Shohei Yamaguchi, Hiroyuki Orita, Naoki Izawa, Saburo Kakizoe, Mototsugu Shimokawa, Tomoharu Yoshizumi, Yoshihiro Kakeji, Masaki Mori & Eiji Oki

Corresponding author:

Hiroshi Saeki, MD, PhD,
Department of General Surgical Science, Gunma University Graduate School of Medicine
E-mail: h-saeki@gunma-u.ac.jp

Online Resource Electronic Supplemental Table 3. Clinical responses in the full analysis set (FAS) study population (n=10)

Clinical Response	n (%)	95%CI
Complete response (CR)	1(10.0)	
Partial response (PR)	6(60.0)	
Stable disease (SD)	2(20.0)	
Unknown	1(10.0)	
Response rate (RR)	7(70.0)	34.8–93.3
※Cases with measurable lesions		
